# Dissociable effects of the prodrug phendimetrazine and its metabolite phenmetrazine at dopamine transporters

**DOI:** 10.1038/srep31385

**Published:** 2016-08-12

**Authors:** Ernesto Solis, Julie A. Suyama, Matthew F. Lazenka, Louis J. DeFelice, S. Stevens Negus, Bruce E. Blough, Matthew L. Banks

**Affiliations:** 1 Department of Physiology and Biophysics, Virginia Commonwealth University, Richmond, VA, 23298 USA; 2 Department of Pharmacology and Toxicology, Virginia Commonwealth University, Richmond, VA, 23298 USA; 3 Institute for Drug and Alcohol Studies, Virginia Commonwealth University, Richmond, VA, 23298 USA; 4 Center for Drug Discovery, Research Triangle Institute, Research Triangle Park, NC, 27709 USA

## Abstract

Phendimetrazine (PDM) is a clinically available anorectic and a candidate pharmacotherapy for cocaine addiction. PDM has been hypothesized to function as a prodrug that requires metabolism to the amphetamine-like monoamine transporter substrate phenmetrazine (PM) to produce its pharmacological effects; however, whether PDM functions as an inactive prodrug or has pharmacological activity on its own remains unclear. The study aim was to determine PDM pharmacological mechanisms using electrophysiological, neurochemical, and behavioral procedures. PDM blocked the endogenous basal hDAT (human dopamine transporter) current in voltage-clamped (−60 mV) oocytes consistent with a DAT inhibitor profile, whereas its metabolite PM induced an inward hDAT current consistent with a DAT substrate profile. PDM also attenuated the PM-induced inward current during co-application, providing further evidence that PDM functions as a DAT inhibitor. PDM increased nucleus accumbens dopamine levels and facilitated electrical brain stimulation reinforcement within 10 min in rats, providing *in vivo* evidence supporting PDM pharmacological activity. These results demonstrate that PDM functions as a DAT inhibitor that may also interact with the pharmacological effects of its metabolite PM. Overall, these results suggest a novel mechanism for PDM therapeutic effects via initial PDM DAT inhibition followed by PM DAT substrate-induced dopamine release.

Phendimetrazine (PDM) is a clinically available and widely prescribed anti-obesity medication^1^. Recently, 14–21 day PDM treatment was shown to decrease cocaine self-administration in preclinical cocaine addiction models^2^,^3^, and it is currently being evaluated as a candidate pharmacotherapy in human laboratory cocaine self-administration studies (NCT02233647 and NCT0252235). Structurally, PDM is an *N*-methyl analog of the monoamine transporter substrate phenmetrazine (PM; Fig. 1) and is hypothesized to function as a prodrug that requires biotransformation to PM as its active metabolite to produce its effects^4^,^5^. From a clinical therapeutic perspective, prodrugs are advantageous for multiple reasons^6^. As one example, prodrug formulations may slow onset of central nervous system-active drug effects reducing abuse liability^7^,^8^. A recent example is the amphetamine prodrug lisdexamfetamine^9^,^10^,^11^. However, research regarding whether PDM functions as an inactive prodrug or has pharmacological activity on its own has produced inconsistent results.

There are two main lines of evidence supporting the hypothesis that PDM functions as an inactive prodrug. First, *in vitro* studies found that PDM possessed measurable but very low potency to block the dopamine transporter (DAT) in rat brain synaptosomes (IC_50_ = 19 μM)^5^. By contrast, PM was approximately 30-fold more potent to block DAT (IC_50_ = 0.6 μM) and 100-fold more potent than PDM to induce release at DAT (EC_50_ = 0.13 μM). Second, *in vivo* microdialysis studies found that PDM failed to significantly increase extracellular nucleus accumbens (NAc) dopamine (DA) levels when either retrodialyzed at a 5-fold larger concentration than its IC_50_ value or administered intravenously at a dose of 10 mg/kg^5^. Conversely, PM increased NAc DA by retrodialysis and after intravenous doses as low as 1 mg/kg. Overall, this literature body suggests that PDM functions as an inactive prodrug for the active metabolite PM.

However, more recent data suggest that PDM may be behaviorally active *in vivo*. Specifically, coordinated pharmacokinetic/pharmacodynamic studies in rhesus monkeys compared the time courses of PDM to be metabolized to PM and to produce cocaine-like discriminative stimulus effects. Notably, the onset of cocaine-like discriminative stimulus occurred within 10 min, a time when PDM plasma levels were high but PM levels were too low to account for the behavioral effects^4^. We interpreted these results to suggest that PDM possesses pharmacological activity in monkeys independent of its metabolism to PM. To further evaluate the potential pharmacological activity of PDM, we aimed to elucidate whether PDM possessed pharmacological activity at DAT with a suitable heterologous system, the two-electrode voltage-clamp technique in *Xenopus laevis* oocytes overexpressing hDAT^12^. Furthermore, given recent evidence demonstrating stereospecificity as a determinant of monoamine transporter inhibitor and substrate pharmacology^13^,^14^,^15^, we also determined the electrophysiological activity of two PDM and PM enantiomers at DAT. Lastly, PDM neurochemical and behavioral effects were determined in rats using *in vivo* microdialysis and intracranial self-stimulation methodologies. These two *in vivo* procedures allow for assessment of the potency and time course of drug effects involving the mesolimbic dopamine pathway^16^,^17^. We hypothesized that PDM would produce *in vitro* electrophysiological effects at DAT and rapid onset of *in vivo* neurochemical and behavioral effects consistent with pharmacological activity independent of its metabolism to PM.

## Results

### Electrophysiological effects of phenmetrazine and phendimetrazine

**Figure 2:**
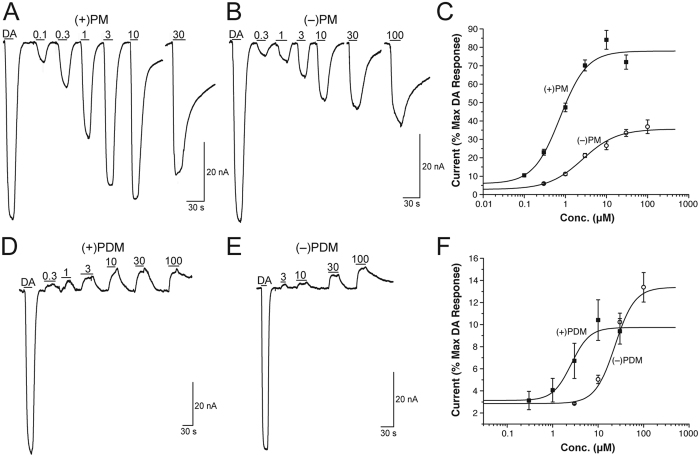
Dose-response of currents induced by phenmetrazine (PM; Panels A,B) and phendimetrazine (PDM; Panels D,E) enantiomers at hDAT in voltage-clamped (−60 mV) *Xenopus laevis* oocytes. Panels C,F show fits of the dose-response function for PM and PDM enantiomers, respectively. Amplitude values are expressed as a % of the amplitude of an initial 5 μM dopamine (DA) effect. All points in Panels C,F represent mean ± SEM data from 5–10 oocytes. Data shown in panels A,B,D,E are from representative current traces.

**Figure 3:**
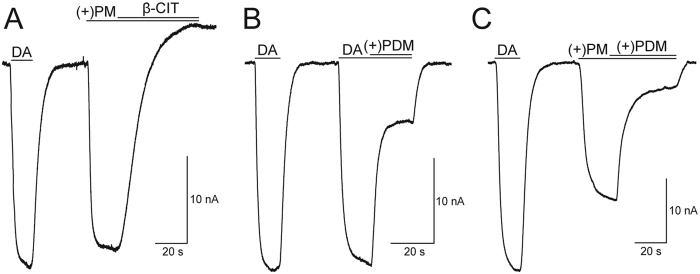
(+)-Phendimetrazine (PDM) effects on either dopamine- (DA) or (+)-phenmetrazine (PM) -induced inward currents at hDAT in *Xenopus laevis* oocytes voltage-clamped to −60 mV. Panel A shows application of 1 μM β-CIT (positive control) blocked the hDAT current induced by 5 μM (+)-phenmetrazine. Panel B shows application of 100 μM (+)-PM attenuated the current induced by 2 μM (+)-PM. Panel C shows application of 100 μM (+)-PDM also attenuated the current induced by 2 μM DA. For all panels, the initial DA-induced response was 5 μM DA.

In recent studies, we have established the two-electrode voltage-clamp technique in *Xenopus laevis* oocytes as a suitable tool to study the interaction of compounds with monoamine transporters^12^,^18^,^19^. Thus, we measured currents in voltage-clamped (−60 mV) oocytes overexpressing hDAT in response to PM and PDM enantiomers. Figure 2 shows PM and PDM enantiomer dose-response functions at hDAT. Both PM enantiomers elicited concentration-dependent inward currents indicating that PM functions as a hDAT substrate (Fig. 2A,B). In contrast, both PDM enantiomers produced concentration-dependent outward currents (representing the block of the endogenous transporter leak current) indicating that PDM functions as an hDAT inhibitor (Fig. 2D,E). The *2S*,*3S*(+)-PM and *2S*,*3S*(+)-PDM enantiomers were nearly 4- and 9-fold more potent than their respective *2R*,*3R*(−) enantiomers (Fig. 2C,F, and Table 1), and the *2S*,*3S* (+)- and *2R*,*3R*(−)-PM enantiomers were 3.4- and 9-fold more potent than their respective PDM enantiomers. To show the specificity of *2S*,*3S*(+)-PM at DAT, Fig. 3A shows application of the DAT inhibitor 2β-carbomethoxy-3β-(4-iodophenyl)tropane (β-CIT; positive control) during *2S*,*3S*(+)-PM application blocked the *2S*,*3S*(+)-PM effect and produced a hyperpolarizing current. To further clarify PDM as a DAT inhibitor, drug-drug interaction studies were conducted to determine whether PDM would block the inward current produced by either dopamine (DA) or *2S*,*3S*(+)-PM similar to β-CIT. Application of 100 μM *2S*,*3S*(+)-PDM during either DA (Fig. 3B) or 2S,3S(+)-PM (Fig. 3C) application attenuated the inward current produced by either compound, providing further evidence that *2S*,*3S*(+)-PDM functioned as an hDAT blocker. Furthermore, a higher *2S*,*3S*(+)-PDM concentration (200 μM) completely blocked the *2S*,*3S*(+)-PM-induced inward current (Supplemental Fig. 1). Supplemental Fig. 2 shows that *2S*,*3S*(+)-PDM is substantially more effective at attenuating the inward current produced by DA at hDAT than *2R*,*3R*(−)-PDM, which is in agreement with the potencies at hDAT alone (Fig. 2).

### *In vivo* neurochemical effects of phendimetrazine

**Figure 4:**
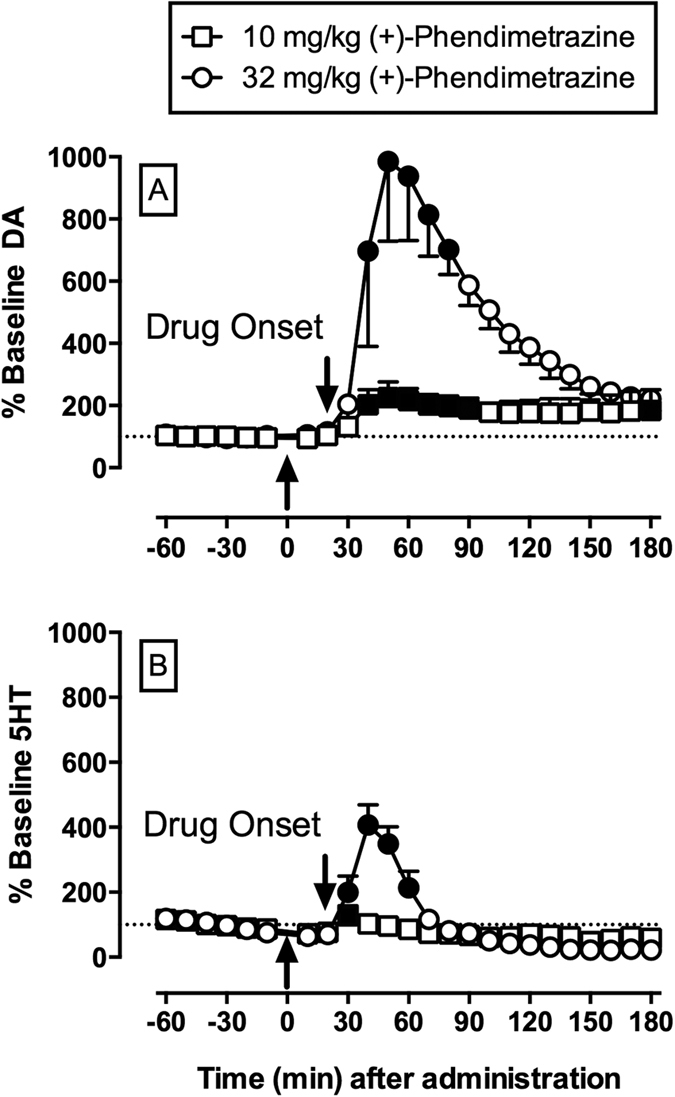
(+)-Phendimetrazine (PDM; 10–32 mg/kg, ip) effects on NAc dopamine (DA) and serotonin (5-HT) levels expressed as a percentage of baseline neurotransmitter levels. Top panel indicates temporal changes in % baseline DA, while bottom panel indicates changes in % baseline 5-HT. Upward arrow indicates time of drug administration. Downward arrow indicates onset of drug effect. Filled symbols indicate statistical significance (p < 0.05) compared to 10 min monoamine levels within a drug dose. All points show mean ± SEM for 4 rats.

Baseline (mean ± SEM) NAc extracellular DA and 5-HT levels were 1.05 ± 0.61 nM and 0.36 ± 0.15 nM, respectively across all microdialysis experiments. Supplemental Fig. 3 shows microdialysis probe placements for all rats included in data analyses. Both *2S*,*3S*(+)-PDM doses significantly increased extracellular NAc DA [10 mg/kg: F_17,51_ = 6.3, p = 0.0037; 32 mg/kg: F_17,51_ = 5.6, p < 0.0001] and 5-HT [10 mg/kg: F_17,51_ = 4.6, p < 0.0001; 32 mg/kg: F_17,51_ = 24.4, p < 0.0001] concentrations and produced sequential periods of rising, peak, and declining monoamine levels during the 180 min sampling period (Fig. 4). Furthermore, both *2S*,*3S*(+)-PDM doses produced greater increases in DA than 5-HT. We should reemphasize that channeling samples directly from the probe to the HPLC system via an autoinjector imposed a 20 min lag time between sample collection and analysis. As a result, the 30-min time point represented the earliest opportunity for drug injection to affect NAc neurotransmitter levels.

### *In vivo* behavioral effects of phendimetrazine

**Figure 5:**
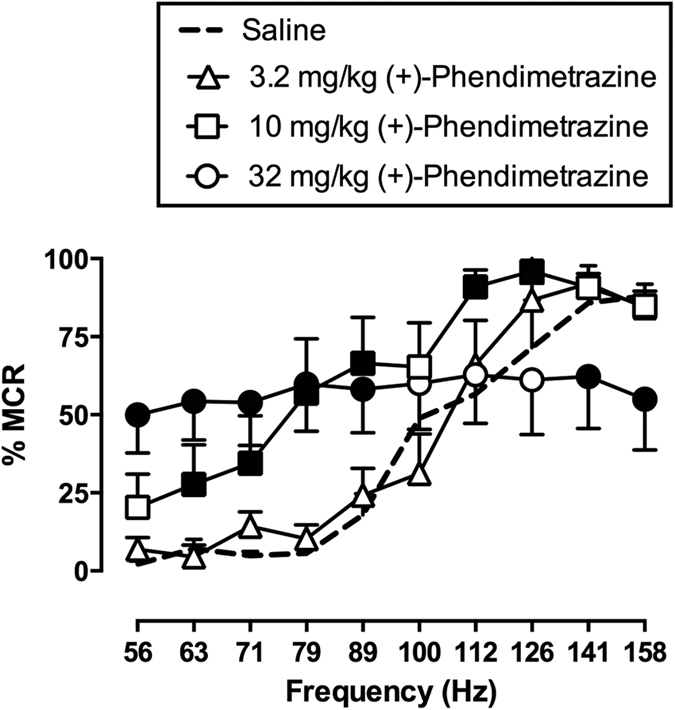
(+)-Phendimetrazine (PDM; 3.2–32 mg/kg, ip) effects on intracranial self-stimulation (ICSS) at 10 min. *Abscissa*: frequency of electrical brain stimulation in Hz. *Ordinate*: ICSS rate maintained by each brain stimulation frequency, expressed as percent maximum control reinforcement rate (%MCR). Filled points represent frequencies at which ICSS rates were statistically different from vehicle rates (p < 0.05). All data show mean ± SEM for 6 rats.

For all six rats in the ICSS experiments, amplitudes ranged from 100 to 195 μΑ, the mean ± SEM baseline maximum control reinforcement rate (MCR) was 57 ± 5 stimulations per trial, and the mean ± SEM number of total baseline stimulations was 226 ± 28 stimulations per component. After saline (vehicle) administration, brain-stimulation reinforcement maintained a frequency-dependent increase in ICSS rates (Fig. 5), and frequency-rate functions after saline administration were similar to baseline performance before saline administration (data not shown). Figure 5 shows *2S*,*3S*(+)-PDM effects (3.2–32 mg/kg) at the 10 min time point as this represented the time of peak effects. *2S*,*3S*(+)-PDM effects on ICSS frequency-rate functions at 30, 100, and 300 min are shown in Supplemental Fig. 3. *2S*,*3S*(+)-PDM produced dose-dependent and biphasic effects on the frequency-rate function with 10 mg/kg exclusively increasing ICSS rates and 32 mg/kg simultaneously increasing low ICSS rates maintained by low brain-stimulation frequencies (56–89 Hz) and depressing high ICSS rates maintained by high brain-stimulation frequencies (158–141 Hz) [frequency × dose: F_27,135_ = 7.46, p < 0.0001]. Rate-increasing effects of 10 mg/kg *2S*,*3S*(+)-PDM were no longer apparent at 300 min. For the larger dose of 32 mg/kg *2S*,*3S*(+)-PDM, rate-increasing effects were sustained throughout the 300-min testing period, but rate-decreasing effects had a shorter duration of action and had largely dissipated at 100 min.

## Discussion

The present study aim was to determine whether PDM possessed pharmacological activity at DAT that might contribute to its therapeutic effects as a candidate anti-cocaine addiction medication. There were two main findings. First, both PDM and PM enantiomers produced an electrophysiological profile at hDAT consistent with PDM being a DAT inhibitor and PM being a DAT substrate. Furthermore, PDM application attenuated the electrophysiological effects of both DA and PM providing further evidence for DAT inhibition properties. Second, PDM produced both *in vivo* neurochemical and behavioral abuse-related effects within 10 min of administration and consistent with the electrophysiology data, demonstrate PDM functions as a DAT inhibitor. Overall, these results suggest PDM possesses a novel pharmacological mechanism of initial DAT inhibition followed by DAT substrate effects of the PDM metabolite PM.

PDM potency to inhibit hDAT in oocytes extends previous PDM results at DAT in rat-brain synaptosomes^5^. The present results extend previous findings by demonstrating the *2S*,*3S*(+) enantiomer was 6-fold more potent than the *2R*,*3R*(−) enantiomer to inhibit hDAT. The PDM enantiomer potency difference reported in the present study was similar to the potency difference (~4.5-fold) to produce cocaine discriminative stimulus effects in monkeys^4^. Previous studies have generally reported >200-fold enantiomer potency differences for other DAT inhibitors, such as pyrovalerone^20^, 3,4-methylenedioxypyrovalerone^13^, and cocaine^21^. However, the DAT inhibitor methylphenidate, which also contains two chiral carbons, displays an approximate 2 to 43-fold potency difference at DAT depending upon the enantiomer comparisons^22^,^23^. Overall, the profile of PDM enantiomer effects at hDAT is consistent with pharmacological effects of known DAT inhibitors with two chiral carbons.

The ability of PM to produce an inward depolarizing current at hDAT was consistent with and extends previous PM results demonstrating it functions as a DAT substrate in rat-brain synaptosomes^5^. The PM enantiomer potency difference (~4-fold) in the present study was consistent with previous PM studies^4^,^5^. Furthermore, 10 μM *2S*,*3S*(+)-PM produced the persistent “shelf” current at hDAT recently described^24^, whereas to observe this current with *2R*,*3R*(−)-PM requires 30 μM concentration. These results further support *2S*,*3S*(+)-PM had a stronger effect at hDAT than *2R*,*3R*(−)-PM and are consistent with previous enantiomer differences with the monoamine transporter substrate amphetamine^25^. In summary, the present results provide further evidence supporting the PDM metabolite PM possesses different and contrasting pharmacological mechanisms compared to its parent compound. Moreover, these PDM and PM electrophysiological effects provide a mechanistic and empirical foundation for the interpretation of subsequent *in vivo* neurochemical and behavioral effects.

PDM-induced increases in extracellular nucleus accumbens DA and 5-HT levels were unexpected from previous studies for three main reasons. First, neither 100 μM PDM retrodialyzed nor 3 mg/kg intravenous PDM significantly increased extracellular DA or 5-HT levels^5^. In the present study, 10 mg/kg *2S*,*3S*(+)-PDM was necessary to produce significant increases in extracellular DA levels. Thus, one potential explanation is that Rothman and colleagues did not test a sufficiently large enough PDM dose to detect significant extracellular DA changes. Second and perhaps more unexpected was the significant changes in extracellular 5-HT levels induced by 32 mg/kg *2S*,*3S*(+)-PDM. Previous *in vivo* and *in vitro* PDM results suggested it had no effect at the serotonin transporter (SERT) up to concentrations of 100 μM^5^. However, we^26^ and others^27^ have reported monoamine transporter substrates are less DA-selective than 5-HT-selective *in vivo* compared to *in vitro* DAT vs. SERT selectivity measures and thus the present results extend these previous findings to monoamine transporter inhibitors. Another potential mechanism for the observed PDM-induced NAc 5-HT effects could be related to DA functioning as a SERT substrate^28^. Lastly, PDM produced significant extracellular DA and 5-HT increases within 20 min of drug onset (the 20 min lag time after drug administration due to microdialysis tubing dead space). Previous studies have implicated NAc DA release in the discriminative stimulus effects of cocaine and other abused monoamine transporter inhibitors^29^,^30^ and the present results corroborate this relationship between NAc DA and both discriminative stimulus effects and ICSS facilitating effects. Although rapid metabolism of *2S*,*3S*(+)-PDM to PM within 20 min might account for these neurochemical effects, pharmacokinetic studies in monkeys demonstrated metabolism to PM could not account for PDM effects at 10 min^4^. Moreover, the onset of *2S*,*3S*(+)-PDM effects is consistent with a temporal profile of neurochemical effects from other monoamine transporter inhibitors^31^ and substrates^5^,^26^,^27^. Overall, these *in vivo* neurochemical data provide further evidence of *2S*,*3S*(+)-PDM possessing significant pharmacological activity at monoamine transporters.

*2S*,*3S*(+)-PDM-induced facilitation of ICSS within 10 min of administration provided further behavioral evidence of PDM pharmacological activity independent of its metabolite phenmetrazine. The rapidity of *2S*,*3S*(+)-PDM ICSS effects was also consistent with previous drug discrimination studies in both nonhuman primates^4^ and rats^32^. Comparison of the present *2S*,*3S*(+)-PDM results and previous *2S*,*3S*(+)-PM ICSS results demonstrated two main findings. First, *2S*,*3S*(+)-PDM was 10-fold less potent than *2S*,*3S*(+)-PM to facilitate ICSS^33^ and this potency difference was consistent with previous preclinical drug discrimination studies^4^,^32^,^34^,^35^. In addition, 32 mg/kg *2S*,*3S*(+)-PDM produced abuse-limiting rate-decreasing effects on ICSS whereas no rate-decreasing effects were evident with *2S*,*3S*(+)-PM, up to doses of 10 mg/kg^33^. Thus, in addition to the lower potency compared to PM, the expression of abuse-limiting rate-decreasing effects, presumably mediated by PDM-induced 5-HT increases, contributes to the lower PDM abuse liability. Second, offset of ICSS effects were longer for PDM compared to PM suggesting PDM metabolism to PM prolonged the duration of PDM effects. PDM also produced longer lasting cocaine discriminative stimulus effects compared to PM in monkeys^4^, but not rats^32^. Overall, these behavioral data provide further evidence of *2S*,*3S*(+)-PDM possessing pharmacological activity independent of its metabolism to PM.

Recently, “atypical” DAT ligands have been proposed as candidate medications for cocaine addiction^36^,^37^. PDM may represent a novel and “atypical” DAT pharmacotherapeutic approach for the cocaine addiction treatment via combined DAT inhibition (PDM) and DAT substrate (PM) mechanisms. Although the exact mechanism by which PDM produces its anti-cocaine effects remains to be fully elucidated, the present results suggest one potential mechanistic framework for understanding PDM therapeutic effects.

## Methods

### Electrophysiological studies in *Xenopus laevis* oocytes

Oocytes were harvested and prepared from adult *Xenopus laevis* females following standard procedures^38^. Experimental protocols complied with the Guide for the Care and Use of Laboratory Animals^39^, were approved by the Virginia Commonwealth University Institutional Animal Care and Use Committee, and are reported according to the ARRIVE guidelines^40^. Furthermore, animal facilities were licensed by the United States Department of Agriculture and accredited by the Association for Assessment and Accreditation of Laboratory Animal Care. Stage V-VI oocytes were selected for cRNA injection within 24 h of isolation. Transporter cRNA was transcribed from the pOTV vector using mMessage Machine T7 kit (Ambion Inc., Austin, TX). Oocytes were injected with 21.6–41.4 ng of hDAT cRNA (Nanoject AutoOocyteInjector, Drummond Scientific Co., Broomall, PA) and incubated at 18 °C for 4–12 days in Ringers solution supplemented with sodium pyruvate (550 μg/mL), streptomycin (100 μg/mL), tetracycline (50 μg/mL), and 5% dialyzed horse serum.

Two-electrode voltage-clamp (TEVC) experiments were conducted as previously described^18^,^24^. Electrodes having resistances from 1–5 MΩ were filled with 3 M KCl. *Xenopus laevis* oocytes expressing hDAT were voltage-clamped to −60 mV with a GeneClamp 500 (Axon Instruments, Sunnyvale, CA), and the holding current was recorded using Clampex 10 (Axon Instruments). The extracellular buffer consisted of (in mM): 120 NaCl, 7.5 HEPES, 5.4 KGluconate, and 1.2 CaGluconate, and had a pH of 7.4. The extracellular buffer was perfused at 5 mL/min until stable baseline currents were obtained at room temperature (23–25 °C). Subsequently, a test compound was added to the buffer and the perfusion duration was indicated as a solid horizontal line over the figure traces.

To determine the PDM and PM enantiomer potency (EC_50_) to elicit hDAT responses, the current amplitude values (expressed as a % of the amplitude of an initial 5 μM DA response) were plotted against concentration and fit to the Hill equation, y = V_min_ + (V_max_ − V_min_) * x^n^/(EC_50_^n^ + x^n^), using Origin 8 (OriginLab Corp., Northampton, MA). The V_max_ is the maximal response, V_min_ is the minimum response, x is the concentration tested for a compound, y is the response measured, the EC_50_ is the concentration that yields half-maximal response, and n is the Hill slope parameter. The EC_50_ is calculated as mean ± standard error of the mean (SEM) from 5–10 oocytes (unless otherwise noted).

### *In vivo* neurochemical and behavioral pharmacology studies

#### Subjects

A total of 11 male Sprague Dawley rats (Harlan, Frederick, MD) weighing 310–350 g at the time of surgery were used. All rats had ad libitum access to standard rodent laboratory chow and continuous access to water in their home cage, except during experimental sessions. Rats were individually housed on a 12-h light-dark cycle, and studies were conducted during the light cycle. Experimental protocols complied with the Guide for the Care and Use of Laboratory Animals^39^, were approved by the Virginia Commonwealth University Institutional Animal Care and Use Committee, and are reported according to the ARRIVE guidelines^40^. Furthermore, animal facilities were licensed by the United States Department of Agriculture and accredited by the Association for Assessment and Accreditation of Laboratory Animal Care.

#### Microdialysis surgery and procedure

Guide cannulae (8 mm long, 0.5 mm outer diameter; CXG-8, Eicom, San Diego, CA, USA) were stereotaxically and bilaterally implanted with a termination point 1 mm above the NAc (coordinates: 1.5 mm anterior to bregma, 1.8 mm lateral to midsagittal suture, 6.0 mm ventral to dura) in rats (n = 5) under isoflurane anesthesia as previously described^26^. Guide cannulae were secured to the skull using screws (Plastics One, Inc., Roanoke, VA, USA) and orthodontic resin (Butler Schein, Dublin, OH, USA). A dummy cannula (CXD-8, Eicom) was inserted into each guide cannula to maintain cannula patency. Animals were allowed at least 7 recovery days prior to initiating microdialysis testing.

On test days, rats were briefly anesthetized with 3.0% isoflurane in oxygen, one of the dummy cannula was removed, and a microdialysis probe (10 mm long, CX-I-8-2, Eicom) with a 2 mm artificial cellulose “cuprophan” membrane (50 kDa molecular weight cut-off) at its tip was inserted into an 8 mm guide cannula such that it extended 2 mm beyond the end of the guide cannula and into the NAc. The probe was connected to a two-channel liquid swivel (TCS2–23, Eicom), and the rat was placed into an acrylic experimental cage (30 cm^3^). Microdialysis probes were perfused with artificial cerebrospinal fluid (aCSF; 147 mM NaCl, 2.8 mM KCl, 1.2 mM CaCl_2_, 1.2 mM MgCl_2_) at a rate of 1 μL/min. The mobile phase consisted of 2% methanol (EMD, Gibbstown, NJ, USA), 100 mM phosphate buffer (Sigma Chemicals, St. Louis, MO, USA), 500 mg/L 1-decane sodium sulfonate (TCI America, Montgomeryville, PA, USA), and 50 mg/L EDTA-2Na^+^ (Dojindo Laboratories, Kumamoto, Japan).

Dialysate samples were collected into a 50 μL injector loop at 10-min intervals using an online auto-injector (EAS-20s, Eicom) and immediately analyzed for DA and 5-HT concentrations by high-pressure liquid chromatography (HPLC) coupled to electrochemical detection (HTEC-500, Eicom). DA and 5-HT were separated using a C_18_-reverse phase column (PP-ODS II, Eicom) and were detected using a graphite working electrode and an Ag^+^ vs. AgCl reference electrode with an applied potential of +450 mV. Preliminary experiments conducted by probe immersion into a known standard concentration of DA indicated a lag time of ~20 min for dialysate to traverse the tubing from the probe to the electrochemical detector at the 1 μL/min flow rate. DA and 5-HT were identified according to characteristic retention times of the standard solution, and concentrations were quantified by comparison with peak heights of the standard concentration curve (0.1–100 pg per 10 μL) generated prior to drug administration in each microdialysis experiment. The lower limit of neurotransmitter detection was 0.1 pg. DA and 5-HT levels were determined to be stable after 6 consecutive stable baseline samples were obtained with <25% variability around the running mean of both neurotransmitters. Subsequently, a *2S*,*3S*(+)-PDM dose (10 or 32 mg/kg) was administered by intraperitoneal injection, and dialysate samples were collected for 180 min after drug administration to generate data for each (+)-phendimetrazine dose in four rats. At the completion of all experiments, rats were euthanized with CO_2_, and brains were removed and stored in 10% formalin. Probe placement was verified by visual inspection of cannula tracks in unstained brain sections as described previously^26^,^41^. Only rats with correct probe placements were included in data analyses (no data were discarded due to improper cannula placement).

#### Data analysis

The primary dependent variables were extracellular DA and 5-HT concentrations in each dialysate fraction expressed as a percent of the average of the 6 mean baseline concentrations before drug or vehicle administration for each experiment. The individual normalized DA and 5-HT concentrations were then averaged across rats to yield group mean results for graphical presentation. Results were analyzed for each drug dose using a repeated measures one-way analysis of variance with time as a fixed main effect and subject as the random effect (JMP Pro 12, SAS, Cary, NC). Using this analytical method, within-subject comparisons using the Dunnet post-hoc test were determined between monoamine concentrations at each time point and the 10-min “control” monoamine concentration. This 10-min time point represents a dialysis sample that was collected before drug administration, that had advanced into the cannula-to-injector-loop tubing at the time of drug administration, and that reached the working electrode for analysis after drug injection due to the 20-min lag time of tubing dead space. The criterion for statistical significance was set at the 95% confidence level (p < 0.05).

#### ICSS surgery and training

Rats (N = 6) were anesthetized using 2–3% isoflurane gas, and the cathode (0.25 mm diameter) of a bipolar electrode (Plastics One, Roanoke, VA) was stereotaxically inserted targeting the medial forebrain bundle: 2.8 mm posterior to Bregma, 1.7 mm lateral to the midsaggital line, and 8.8 mm ventral to the skull as described previously^33^,^42^. Three stainless steel screws were placed in the skull, and the anode (0.125 mm diameter, uninsulated) was wrapped around one of these screws to act as a ground. Dental acrylic was used to secure the electrode. Animals were allowed at least 5 recovery days before operant training was initiated. Rats were subsequently trained to respond for brain stimulation (0.5-s train of square-wave cathodal pulses [0.1 ms/pulse]) under a fixed-ratio (FR) 1 schedule of reinforcement using equipment (Med Associates, St. Albans, VT) and procedures as previously described^14^,^33^. During the terminal ICSS procedure, brain stimulation was available during three consecutive daily components, and each 10 min component consisted of 10 1-min trials. The available stimulation frequency for the first trial was 158 Hz, and frequency decreased by 0.05 log units during each of the subsequent 9 trials to a final frequency of 56 Hz. Each trial started with a 10 s time-out period, during which responding had no scheduled consequences, and 5 non-contingent stimulations at the designated frequency were delivered at one second intervals during the last 5 s of the time out. During the remaining 50 s of each trial, responding produced both intracranial stimulation at the designated frequency and illumination of the lever lights. Stimulation amplitude was individually adjusted to identify an amplitude that maintained greater than half-maximal responding at the 5 highest frequencies. Once this amplitude was identified, training continued until group mean frequency-rate curves were not statistically different over three consecutive training days (see Data Analysis).

#### Phendimetrazine effects on ICSS

Time course test sessions consisted of three consecutive baseline components followed first by IP administration of saline (vehicle) or *2S*,*3S*(+)-PDM (3.2–32 mg/kg) and then by pairs of consecutive test components beginning 10, 30, 100, and 300 min after saline or *2S*,*3S*(+)-PDM administration. Test sessions were usually conducted on Tuesdays and Fridays, and three-component baseline training sessions were conducted on Mondays, Wednesdays, and Thursdays.

#### Data Analysis

The primary dependent measure was reinforcer rate in stimulations per minute during each frequency trial. Raw reinforcer rates from each trial were converted to percent maximum control rate (%MCR), with maximum control rate defined as the mean of the maximal rates obtained at any frequency during the second and third baseline components for that day in that rat. Thus, %MCR values for each trial were calculated as (reinforcement rate during a frequency trial ÷ MCR) × 100. For each test session, data from the second and third baseline components were averaged to yield a baseline frequency–rate curve, and data from test components were averaged to generate test frequency–rate curves. Baseline and test curves were then averaged across rats to yield mean baseline and test curves for each manipulation. Group mean frequency-rate curves were analyzed by repeated measures two-way ANOVA with ICSS frequency and drug dose as factors. A significant ANOVA was followed by the Holm-Sidak multiple comparisons *post-hoc* test. As a summary measure of drug effects, the total number of stimulations per component was also calculated and converted to percent total baseline stimulations per component. Test data were normalized to individual baseline data expressed as percent baseline total stimulations per component = (mean total stimulations per test component)/(mean total stimulations per baseline component) × 100. Data were then averaged across rats for each drug dose. The criterion for statistical significance was set at the 95% confidence level (*P* < 0.05). All analyses were conducted using Prism 6.0c for Mac (GraphPad Software, La Jolla, CA).

#### Drugs

(+)-Phenmetrazine {(*2S*,*3S*)-(+)-3-Methyl-2-phenylmorpholine} fumarate, (−)-phenmetrazine {(*2R*,*3R*)-(+)-3-Methyl-2-phenylmorpholine} fumarate, (+)-phendimetrazine {(*2S*,*3S*)-(+)-3,4-Dimethyl-2-phenylmorpholine} hemifumarate and (−)-phendimetrazine {(*2R*,*3R*)-(+)-3,4-Dimethyl-2-phenylmorpholine} hemifumarate were synthesized by BE Blough (RTI, Research Triangle Park, NC). β-CIT (2β-carbonmethoxy-3β-(4-iodophenyl)tropane; aka RTI-55) was a gift from Habibeh Khoshbouei. For the oocyte studies, all drugs were dissolved in the extracellular solution described above. For the microdialysis and ICSS studies, *2S*,*3S*(+)phendimetrazine hemifumarate was dissolved in sterile saline and administered intraperitoneally in a volume of 1 mL/kg. All concentrations and doses were expressed as the salt forms listed above.

## Additional Information

**How to cite this article**: Solis, E., Jr. *et al*. Dissociable effects of the prodrug phendimetrazine and its metabolite phenmetrazine at dopamine transporters. *Sci. Rep.* **6**, 31385; doi: 10.1038/srep31385 (2016).

## Supplementary Material

Supplementary Information

## Figures and Tables

**Figure 1:**
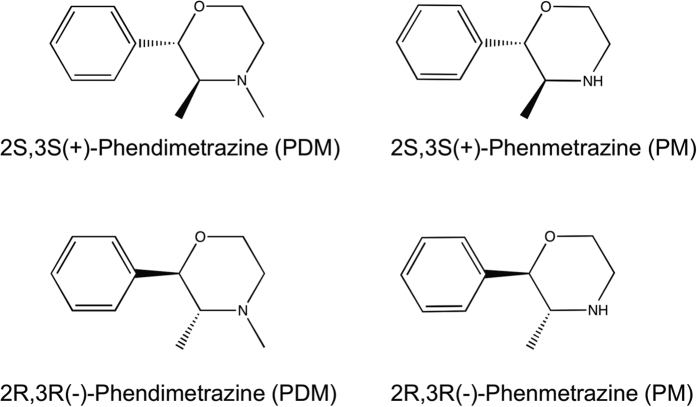
Chemical structure of phendimetrazine and phenmetrazine enantiomers examined in the present study.

**Table 1:** Mean EC_50_ values for phendimetrazine (PDM) and phenmetrazine (PM) enantiomers to produce either a hyperpolarizing (PDM) or inward (PM) current in oocytes expressing hDAT.

Compound	EC_50_ ± SEM (μM) values
*2S*,*3S*(+)-Phendimetrazine	2.56 ± 0.82
*2R*,*3R*(−)-Phendimetrazine	23.5*
*2S*,*3S*(+)-Phenmetrazine	0.75 ± 0.14
*2R*,*3R*(−)-Phenmetrazine	2.63 ± 0.60

^*^Estimated EC_50_ value.
