# Supplementary material for: Dissociable effects of the prodrug phendimetrazine and its metabolite phenmetrazine at dopamine transporters
Source: Sci Rep. 2016 Aug 12;6:31385. doi: 10.1038/srep31385 (PMC4981850; doi:10.1038/srep31385)
Supplement: Supplementary Information [file srep31385-s1.pdf]

Supplemental Materials

Dissociable effects of the prodrug phendimetrazine and its metabolite phenmetrazine at dopamine transporters

Ernesto Solis Jr[1], Julie A Suyama[2], Matthew F Lazenka[2], Louis J De Felice[1], S Stevens Negus[2,3], Bruce E Blough[4], Matthew L Banks[2,3*]

[1]Department of Physiology and Biophysics, Virginia Commonwealth University, Richmond, VA, USA 23298

[2]Department of Pharmacology and Toxicology, Virginia Commonwealth University, Richmond, VA, USA 23298

[3]Institute for Drug and Alcohol Studies, Virginia Commonwealth University, Richmond, VA, USA 23298

[4]Center for Drug Discovery, Research Triangle Institute, Research Triangle Park, NC, USA 27709

Number of Figures: 4

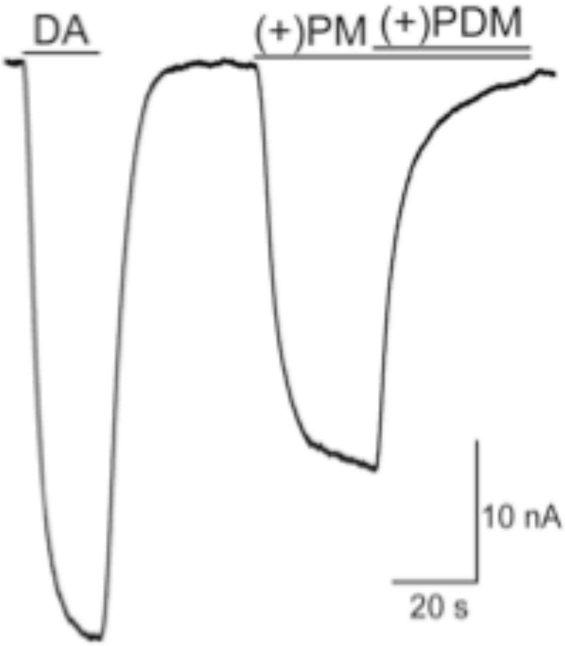

Figure 1: (+)-Phendimetrazine (PDM) effects on (+)-phenmetrazine (PM) -induced inward currents at hDAT in *Xenopus laevis* oocytes voltage-clamped to –60 mV. The initial DA-induced response was 5 µM DA. This representative trace shows application of 200 µM (+)-PDM attenuated the current induced by 2 µM (+)-PM.

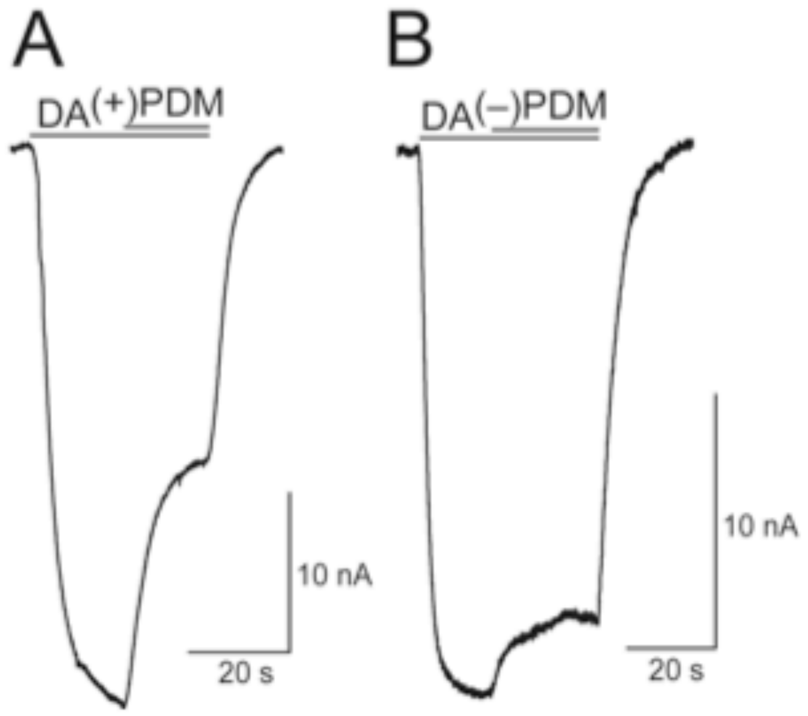

Figure 2: (+)-Phendimetrazine (PDM) and (−)-PDM effects on dopamine- (DA) -induced inward currents at hDAT in *Xenopus laevis* oocytes voltage-clamped to –60 mV. Panel A shows application of 50 µM (+)-PDM attenuated the current induced by 5 µM DA. Panel B shows application of 100 µM (−)-PDM attenuated the current induced by 5 µM DA; however, this effect was weaker than (+)-PDM due to the lower potency of (−)-PDM.

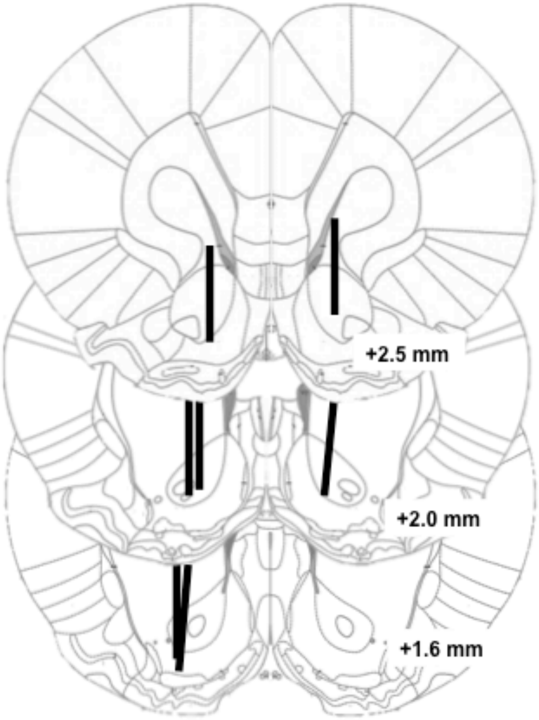

Figure 3: Coronal section depiction of the rat brain (adapted by authors from Paxinos and Watson, 2006) showing the positions of the microdialysis probes. Number in the bottom right indicates anterior-posterior position relative to bregma.

Paxinos, G. and Watson, C. *The Rat Brain in Stereotaxic Coordinates*, 6th edition, (Academic Press, San Diego 2006).

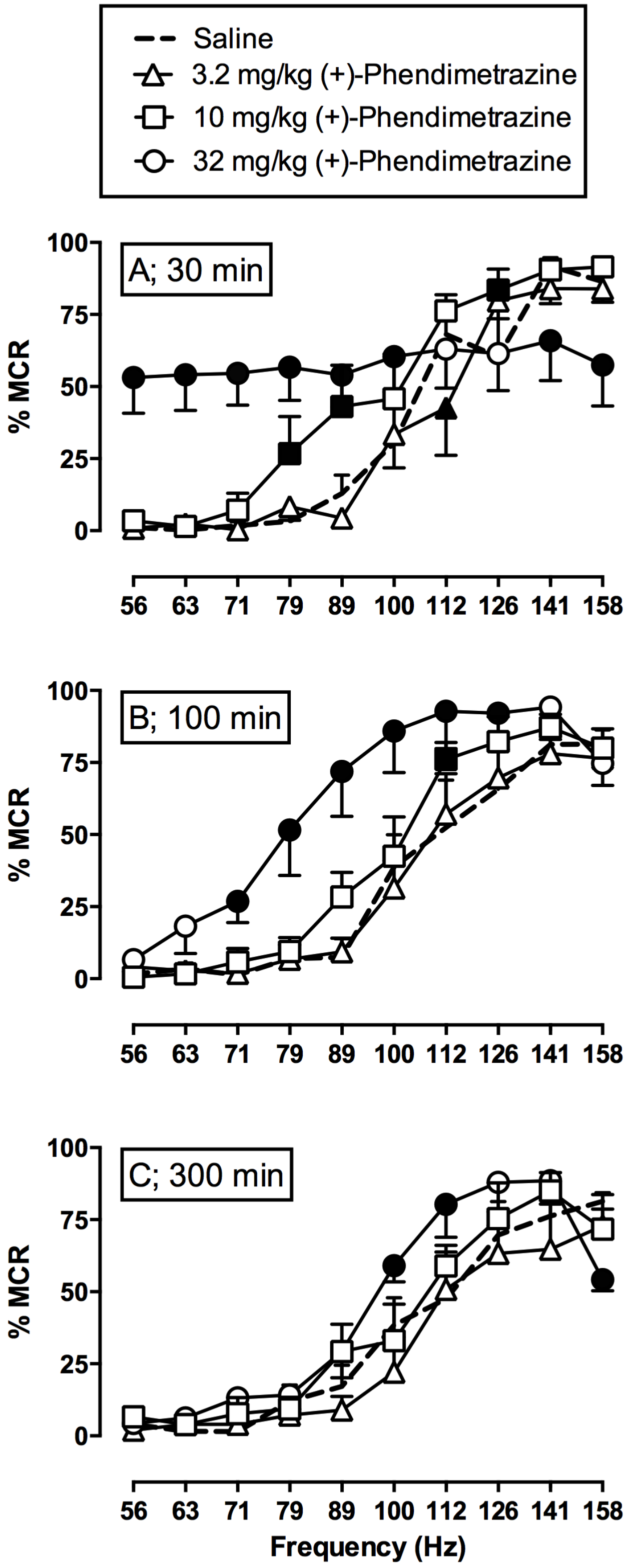

Saline
3.2 mg/kg (+)-Phendimetrazine
10 mg/kg (+)-Phendimetrazine
32 mg/kg (+)-Phendimetrazine
A; 30 min
B; 100 min
C; 300 min
% MCR
100
75
50
25
0
56
63
71
79
89
100
112
126
141
158
Frequency (Hz)

Figure 4: (+)-Phendimetrazine (PDM; 3.2-32 mg/kg, ip) effects on intracranial self-stimulation (ICSS) at 30 min (Panel A), 100 min (Panel B), and 300 min (Panel C). *Abscissa*: frequency of electrical brain stimulation in Hz. *Ordinate*: ICSS rate maintained by each brain stimulation frequency, expressed as percent maximum control reinforcement rate (%MCR). Filled points represent frequencies at which ICSS rates were statistically different from vehicle rates ($p<0.05$). All data show mean ± SEM for 6 rats. 30 min (frequency: $F_{9,45}=49.3$, $p<0.0001$; dose × frequency interaction: $F_{27,135}=8.0$, $p<0.0001$). 100 min (frequency: $F_{9,45}=51.7$, $p<0.0001$; dose: $F_{3,15}=4.8$, $p=0.0152$; dose × frequency interaction: $F_{27,135}=2.6$, $p=0.0002$). 300 min (frequency: $F_{9,45}=52.5$, $p<0.0001$; dose: $F_{3,15}=7.1$, $p=0.0034$; dose × frequency interaction: $F_{27,135}=1.9$, $p=0.0071$).
